# Inflammatory Myofibroblastic Tumor of the Thigh: Presentation of a Rare Case and Review of the Literature

**DOI:** 10.1155/2015/814241

**Published:** 2015-04-05

**Authors:** O. D. Savvidou, V. I. Sakellariou, O. Papakonstantinou, E. Skarpidi, P. J. Papagelopoulos

**Affiliations:** 1st Department of Orthopaedics, Athens University Medical School, Attikon University General Hospital, 1 Rimini Street, Chaidari, 12462 Athens, Greece

## Abstract

Inflammatory myofibroblastic tumors are uncommon neoplasms; presentation of these tumors in the lower extremities is extremely rare. We present a case of a 47-year-old male with fever, fatigue, and a slow-growing thigh mass. The inflammatory markers were elevated and the MR images showed a well-defined intermuscular lesion with mild heterogeneous enhancement. The lesion was excised and histologic examination was consistent with an inflammatory myofibroblastic tumor. No adjuvant therapy was needed and the patient remained asymptomatic with no evidence of tumor recurrence during the 2 years of follow-up.

## 1. Introduction

Inflammatory myofibroblastic tumours (IMT) have emerged from a broad category of inflammatory pseudotumours, with distinctive clinical, pathological, and molecular features [1]. According to the World Health Organization IMTs are classified as tumors of intermediate biological potential due to a tendency for local recurrence; however they rarely metastasize [2–5].

In the past, these tumors were referred as plasma cell granulomas, xanthomatous pseudotumors, pseudosarcomatous myofibroblastic proliferations, myofibroblastomas, inflammatory myofibrohistiocytic proliferations, and inflammatory pseudotumors [6–8]. They usually occur in the lungs [9–11]. Although IMTs have been reported in various extrapulmonary sites, its occurrence in lower extremity is extremely rare [9, 10, 12–14].

In this paper we aimed to present the unique clinical and radiographic features of this rare tumor, the differential diagnosis and treatment options through a review of the current literature.

## 2. Case Presentation

A 47-year-old male was referred to our hospital due to persisting fatigue and low-grade fever that lasted for the past 4 months. A slow-growing painful mass located at the left thigh was also noted.

Blood analysis showed a severe hypochromia, microcytic anemia (HCT: 18.2%, HGB: 5.4 g/dL, MCV: 53.4 Fl, MCH: 15.8 pg, and MCHC: 29.6 g/dL), and thrombocytosis (PLT: 705 × 103/*μ*L). The white blood cell count (WBC) was normal. On admission laboratory tests revealed markedly elevated inflammatory markers: C-reactive protein (CRP) was 152 mg/dL and erythrocyte sedimentation rate (ESR) 112 mm/h. Mantoux and Vidal-Wright tests were negative.

Physical examination revealed a moderately solid and firm deep, stack mass located proximally at the anterior-medial aspect of the left thigh. The size of the mass was measured approximately 8 × 6 cm. There was no history of trauma.

**Figure 1:**
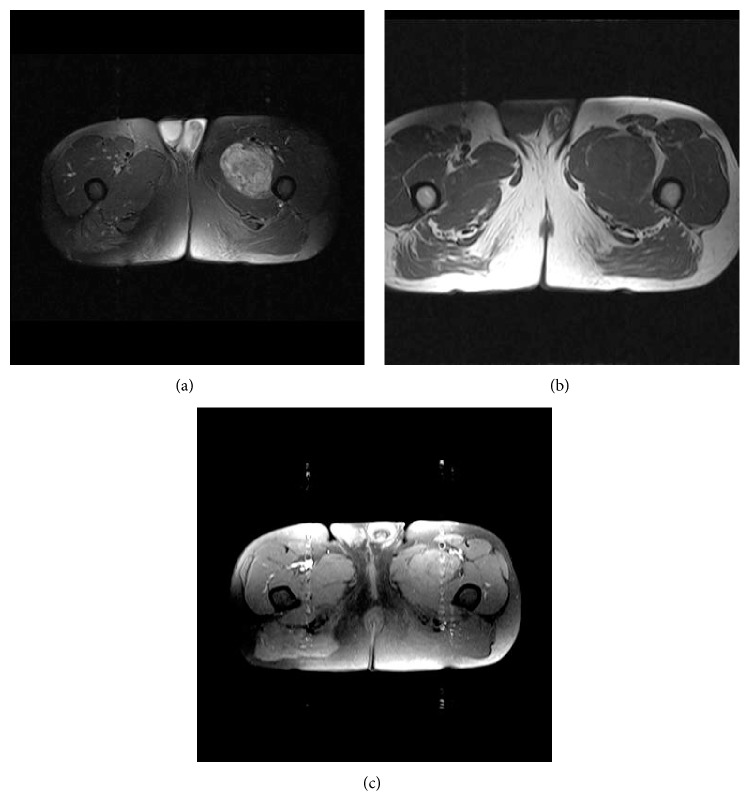
(a) Axial T1-weighted TSE image shows a hypointense intermuscular mass. (b) The mass is inhomogenously hyperintense on an axial T2-weighted TSE fat suppressed image. (c) Axial T1-weighted TSE image with fat suppression after intravenous administration of gadolinium exhibits faint in homogenous enhancement of the lesion.

Radiographically, the mass had no calcifications. The chest X-ray was normal. Magnetic resonance imaging (MRI) of the thigh demonstrated a well-circumscribed mass, located in the intermuscular space between the vastus medialis, the vastus intermedius, and the adductor muscles. The mass was measured 8.5 × 6.6 × 5.8 cm. The lesion was characterized by low signal intensity on the T1-weighted images (Figure 1(a)); heterogeneous high signal intensity on T2-weighted images with fat suppression (Figure 1(b)) and mild enhancement on T1 contrast enhanced MR images (Figure 1(c)). Multiple hypointense spots within the mass were apparent in all sequences and were attributed in foci of hemosiderin deposition.

A CT-guided biopsy showed a moderately cellular fibroblastic/myofibroblastic lesion with features suggestive of inflammatory myofibroblastic tumor. The differential diagnosis included a low grade fibroblastic/myofibroblastic sarcoma and a low grade myxofibrosarcoma.

**Figure 2:**
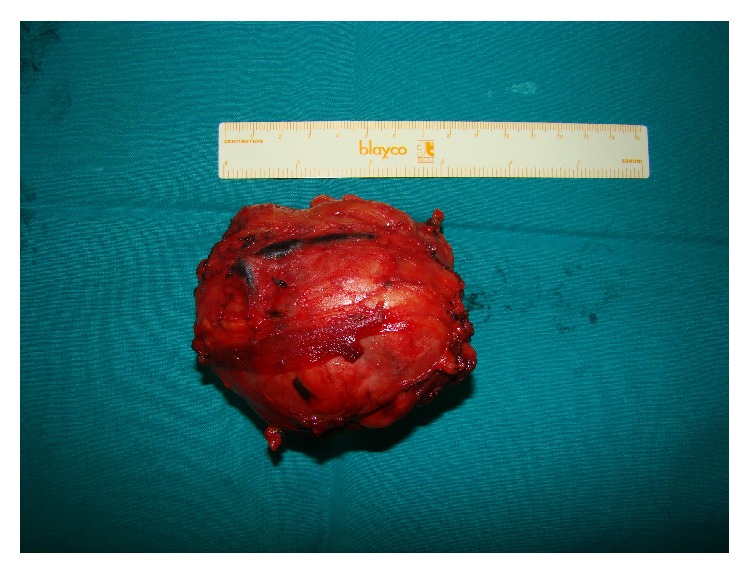
Gross specimen consisting of an ovoid tumor with a firm, fibroelastic white, tan cut surface.

**Figure 3:**
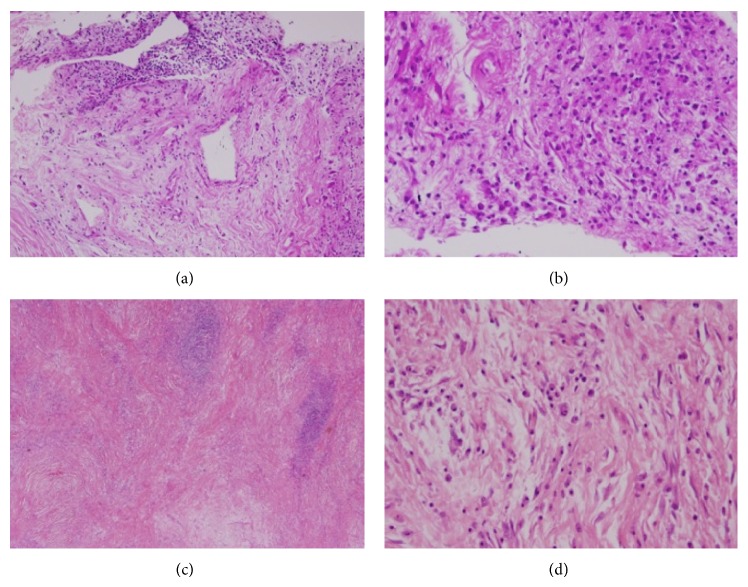
Microscopic examination showing a variably cellular tumor consisting of hypocellular collagenized fibrous areas and intervening myxoid areas (a, c) with mildly atypical spindle cells and dense inflammatory infiltrates of lymphocytes and plasma cells (b, d).

Excisional biopsy was performed and the mass was resected en bloc. The gross specimen consisted of an ovoid tumor with a firm, fibroelastic white, tan cut surface (see Figure 2). The microscopic examination showed a variably cellular tumor consisting of hypocellular collagenized fibrous areas and intervening myxoid areas with mildly atypical spindle cells and dense inflammatory infiltrates of lymphocytes and plasma cells (Figure 3). No significant mitotic activity or necrosis was observed.

**Figure 4:**
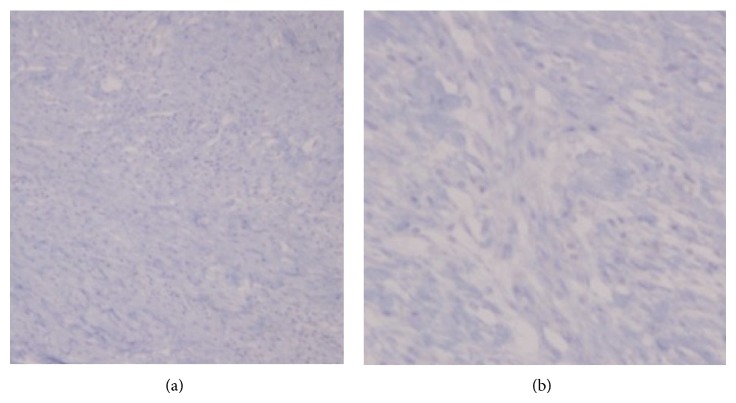
Absence of ALK expression by immunohistochemical staining ((a): ×100, (b): ×200).

Immunohistochemical examination showed the spindle cells to be variably positive for CD68 and focally positive for SMA and calponin expression while they were negative for CKAE1/AE3, S-100 protein, desmin, EMA, ALK, CD34 and MDM2 expression (Figure 4). Immunohistochemical examination for *κ*- and *λ*-immunoglobulin light chain expression was consistent with a polyclonal plasma cell population.

Postoperatively, the clinical signs and symptoms regressed, while laboratory tests gradually normalized. No adjuvant treatment was given. One month later an MRI of the thigh was performed showing absence of residual tissue of the pre-existent tumour. Thus, no further treatment was considered necessary. After 2 years of follow-up, the patient remains asymptomatic with negative laboratory and imaging tests and no signs of recurrence or metastatic disease.

## 3. Discussion

IMTs have emerged from a broad category of inflammatory pseudotumours, with distinctive clinical, pathological and molecular features. Herein, we presented a very rare case of inflammatory myofibroblastic tumor that was developed in a very uncommon anatomic location with a misleading symptomatology. We show the unique clinical and radiographic features of this rare tumor, the pathology and the differential diagnosis, along with the treatment options.

The World Health Organization (WHO) classification of tumors of soft tissue and bone currently defines IMT as a distinctive neoplasm composed of myofibroblastic and fibroblastic spindle cells accompanied by an inflammatory infiltrate of plasma cells, lymphocytes, and/or histiocytes [15, 16]. Historically, IMT was first reported in the lungs in 1937 and was officially included in the World Health Organization classification of soft tissue tumors in 1994 [17–19].

The pathogenesis of IMT remains unclear, although various allergic, immunologic and infectious mechanisms have been postulated [20]. Multiple studies have shown expression of p80 and the clonal rearrangement of the anaplastic lymphoma kinase (ALK) gene on chromosome 2p23 leading to the overexpression of the oncoprotein in the spindle cell components of some of these tumors [21, 22]. Abnormalities in chromosome 2p are seen in up to 60% of patients younger than 10 years of age [21]. This finding indicates a true neoplastic nature for the tumor cells [23]. DNA aneuploidy and association of the lesion with oncogenic viruses such as Epstain-Barr virus, Human Herpes virus type 8 and overexpression of IL-6 have also been demonstrated and proposed to be involved in the pathogenesis of the tumor by some investigators [23, 24].

The lesion usually occurs in children and young adults although it may occur as late as the eighth decade of life [1, 22, 25] without any gender predilection [18, 26]. Gleason and Hornick emphasized that the diagnosis of IMT in middle aged or elderly patients should be made with caution [1] (Table 1).

The lung is the most common site of involvement but the neoplasm has also been reported in other organs including the stomach, mesentery, omentum, and retroperitoneum as well as the kidneys, renal pelvis, liver, spleen, esophagus, and lymph nodes [16, 27]. Pelvis, head and neck, trunk, and extremities are considered uncommon locations for the development of these neoplastic conditions [10, 28].

The biologic potential of IMTs is highly variable. In general, these are classified as tumours of intermediate biological activity with uncertain malignant potential. They may be associated with intermediate incidence of local recurrence, infiltrative growth, vascular invasion, and malignant sarcomatous transformation although these tumors are typically benign [29]. The recurrence rate varies by anatomical site, from <2% for tumours confined to the lung [7, 30] to 25% for extrapulmonary lesions [10]. Several recent studies have reported that recurrence is very infrequent following complete excision of a solitary lesion [7, 18, 31]. Distant metastasis of IMT is rare, occurring in <5% of cases [2].

The clinical presentation of IMT varies depending on the anatomic location and they are frequently misinterpreted. Patients generally present with a mass without specific symptomatology. Vague abdominal pain or gastrointestinal complaints for intra-abdominal lesions, and cough, chest pain, or less often haemoptysis for pulmonary tumours consist the most common symptoms [32]. Fever, weight loss, pain, and malaise are present in 15–30% of patients.

Laboratory evaluation may reveal microcytic anaemia, a raised ESR, thrombocytosis, and/or polyclonal hypergammaglobulinaemia [1, 19, 33]. The production and elevated serum concentrations of interleukin 6 may play a role in diagnosis [34]. The systemic manifestations resolve following surgical excision, and tumor recurrence may be proclaimed by a return of clinical and laboratory abnormalities [10, 27].

Patients with IMTs of the extremities have no systemic symptoms; anemia, fever, or weight loss, and laboratory abnormalities are uncommon [13]. However our patient had high levels of CRP and ESR.

Identification of a soft tissue mass is usually incidental as a result of an extensive workup for fever of unknown origin or growth failure [35]. The MR imaging features of IMT of the thigh are nonspecific on T1 and T2 weighted images. The IMT of the thigh [13] is reported to preferably create a slow growing mass located at the medial aspect of the thigh. In our case, there was a slow-growing mass in the intermuscular interval between adductors and vastus medialis and intermedius. In all cases the MR characteristics of the lesion showed a hypointense mass on T1 and with variable intensity on T2 sequence. Specifically on T2 weighted images the magnetic intensity ranged from hypointense to nonhomogenously hyperintense lesion. Previous reports have shown that there is a moderate to intense enhancement of the lesion with the use of contrast substance; whereas in our case there was only mild enhancement best seen in delayed images, probably due to the presence of abundant fibrous tissue around the lesion. Han and Lin have also noted that the degree of enhancement is associated with the relative cellularity and the amount of fibrous tissue of the myofibroblastic tumor [13, 36]. Delayed and mild enhancement after administration of gadolinium is usually seen in lesions with excessive fibrous component such as desmoids and nodular fasciitis [13, 36]. However, malignant tumors such as MFH (malignant fibrous histiocytoma), and fibro sarcomas should be included in the differential diagnosis. In our case, the age of the patient, the location of the mass, the vagueness of clinical presentation, the fever and fatigue and the nonspecific imaging features contributed not to include IMT in the preoperative diagnosis.

The definite diagnosis relies on histological and immunohistochemical confirmation of the disease. However, tissue samples obtained by CT-guided fine-needle or true-cut biopsies are usually insufficient to establish a definitive histological diagnosis and therefore the final diagnosis relies on the pathologic examination of the surgical resection specimen. Grossly, IMTs may be firm, solid or rubbery, occasionally gelatinous, with a white or tan cut surface. Tumour size ranges from 2 cm to 20 cm, with a mean size of 6 cm. Calcification, haemorrhage and necrosis are identified in a minority of cases [10, 37].

Histologically, IMTs are composed of myofibroblastic and fibroblastic spindle cells with inflammatory infiltrate of lymphocytes, plasma cells, eosinophils, histiocytes in a background of abundant blood vessels. There exists a mixture of three different histopathological patterns. The first resembles a nodular fasciitis with elongated myofibroblasts containing abundant eosinophilic cytoplasm and vesicular nuclei, loose myxoid stroma with neutrophils, lymphocytes, and eosinophils, but few plasma cells. The second pattern is characterized by the presence of cellular with spindled myofibroblasts and fibroblasts in more compact stroma, arranged as islands surrounded by fibromyxoid stroma with prominent plasma cells and mitotic figures. The third pattern has densely hyalinized stroma with few spindle cells, few plasma cells or lymphocytes. All 3 histological patterns have no nuclear pleomorphism and no atypical mitotic figures [38–40].

The differential diagnosis includes benign or malignant spindle-cell tumors such as fibrous histiocytoma, fibroblastic/myofibroblastic tumor and solitary fibrous tumor. They spindle-cell morphology can be misdiagnosed as both benign reactive processes and malignant spindle-cell neoplasms due to their similar morphology. The presence of nuclear hyperchromasia, atypical mitoses, or more than mild nuclear atypia argues strongly against the diagnosis.

From the immunohistochemical stand point, the differential diagnosis includes a variety of neoplastic and reactive lesions. The dedifferentiated liposarcoma can have prominent inflammatory myofibroblastic tumor-like features with expressions of MDM2 and CDK4 for identification [41, 42]. Therefore the presence or absence of a well-differentiated liposarcoma component and expression of MDM2 and CDK4 should be considered in the diagnosis of IMTs and requires further research.

The mainstay of treatment for this tumor is surgical resection with wide margins [2]. Radiotherapy, immunosuppression, and chemotherapy have not been proven to have any definitive benefit. Chemotherapy is useful in cases of multifocal, invasive lesions or in cases of local recurrence. Corticosteroids are generally not useful in adults, although good results have been reported in children in cases of unresectable tumors or hilar and mediastinal invasion [19, 24, 43–45].

Complete removal of the tumor leads to resolution of all symptoms and laboratory abnormalities. After complete resection, the prognosis of IMT is generally good with a low risk of distant metastasis. Incompletely resected tumors may have local recurrence within 1 year [10, 43, 46]. In our patient the tumor was completely resected.

Because IMT exhibits uncertain malignant potential ranging from local recurrence to malignant transformation, it should be closely followed clinically or radiologically over a long-term period. Recurrence has been seen up to 9 years after resection of the primary tumor [47]. Our patient had a complete surgical excision of the tumor. Although the tumor was negative for ALK, the follow-up did not indicate metastatic disease or recurrence.

## Figures and Tables

**Table 1:** Clinical and pathological features helping differential diagnosis of inflammatory myofibroblasic tumor.

Features that favor IMT	Features that argue against IMT
Child or young adult	Middle aged or older adult
Mass in lung or soft tissue of abdomen, pelvis, and retroperitoneum	Mass of the skin or subcutis, lymph nodes, spleen, or bladder
Diffuse inflammatory infiltrate, prominent plasma cells	Patchy inflammatory infiltrate. Predominantly lymphocytic
Mild nuclear atypia, scattered ganglion-like cells	Moderate to severe nuclear atypia with hyperchromasia
Low mitotic rate, no atypical forms	Atypical mitoses
ALK positivity by immunohistochemistry or ALK gene rearrangement	Necrosis
